# Reduced macula microvascular densities may be an early indicator for diabetic peripheral neuropathy

**DOI:** 10.3389/fcell.2022.1081285

**Published:** 2022-12-09

**Authors:** Xiaoyu Deng, Shiqi Wang, Yan Yang, Aizhen Chen, Jinger Lu, Jinkui Hao, Yufei Wu, Qinkang Lu

**Affiliations:** ^1^ The Affiliated People’s Hospital of Ningbo University, Ningbo, China; ^2^ Cixi Institute of Biomedical Engineering, Ningbo Institute of Materials Technology and Engineering, Chinese Academy of Sciences, Ningbo, China

**Keywords:** diabetic retinopathy, microvasculature, optical coherence tomography angiography, vascular length density, diabetic peripheral neuropathy

## Abstract

**Purpose:** To assess the alteration in the macular microvascular in type 2 diabetic patients with peripheral neuropathy (DPN) and without peripheral neuropathy (NDPN) by optical coherence tomography angiography (OCTA) and explore the correlation between retinal microvascular abnormalities and DPN disease.

**Methods:** Twenty-seven healthy controls (42 eyes), 36 NDPN patients (62 eyes), and 27 DPN patients (40 eyes) were included. OCTA was used to image the macula in the superficial vascular complex (SVC) and deep vascular complex (DVC). In addition, a state-of-the-art deep learning method was employed to quantify the microvasculature of the two capillary plexuses in all participants using vascular length density (VLD).

**Results:** Compared with the healthy control group, the average VLD values of patients with DPN in SVC (*p* = 0.010) and DVC (*p* = 0.011) were significantly lower. Compared with NDPN, DPN patients showed significantly reduced VLD values in the SVC (*p* = 0.006) and DVC (*p* = 0.001). Also, DPN patients showed lower VLD values (*p* < 0.05) in the nasal, superior, temporal and inferior sectors of the inner ring of the SVC when compared with controls; VLD values in NDPN patients were lower in the nasal section of the inner ring of SVC (p < 0.05) compared with healthy controls. VLD values in the DVC (AUC = 0.736, *p* < 0.001) of the DPN group showed a higher ability to discriminate microvascular damage when compared with NDPN.

**Conclusion:** OCTA based on deep learning could be potentially used in clinical practice as a new indicator in the early diagnosis of DM with and without DPN.

## Introduction

The incidence of diabetes has been rising worldwide in recent years. The number of people diagnosed with diabetes is expected to reach 800 million by 2045 (Sun et al., 2022). Vascular complications (including microvascular and macrovascular) are the main reasons for the increase in morbidity and mortality in diabetic patients (Sarwar et al., 2010; Kim et al., 2021). Long-term hyperglycemia may lead to large and small vessel abnormalities; some of the major complications include cardiovascular disease, diabetic nephropathy, diabetic retinopathy, and neuropathy (Cole and Florez 2020). Although some of the studies showed that intensive blood glucose control could reduce the risk of microvascular complications in diabetic patients, such as diabetic retinopathy (Chew et al.,2010) and diabetic peripheral neuropathy (DPN) (Callaghan et al., 2012), the beneficial effects of intensive control on macrovascular and cardiovascular endpoints in patients with type 2 diabetes are vague. Therefore, early detection of complications related to diabetes and further understanding of their underlying pathology are of crucial importance.

DPN is one of the most common complications of type 1 and type 2 diabetes. More than half of diabetic patients suffer from peripheral nerve injury (Kazamel et al., 2021). DPN mainly leads to chronic neuropathic pain, numbness and tingling of extremities, paresthesia, and foot ulcer. Its diagnosis commonly relies on traditional measurement methods of neuropathy, such as nerve conduction studies, skin biopsy evaluation, Michigan Neuropathy Symptom Inventory (MNSI), and the Utah Early Neuropathy Scale (UENS) (Singleton et al., 2008; Iqbal et al., 2018). Yet, these diagnostic methods have low repeatability, poor sensitivity (especially in detecting early-stage disease), and certain hysteresis in early clinical diagnosis (Malik 2014). If not timely treated, DPN develops into a diabetic foot, leading to amputation. Previous studies have shown that the 5-year survival rate of diabetic patients after amputation is even lower than that of prostate cancer and breast cancer (Selvarajah et al., 2019).

The recent development of new ophthalmological examination methods allows the evaluation of systemic vascular and nerve damage by using the changes in ocular vascular and nerve microstructures. Because the retina is suggested as a site of diabetic damage, prior studies suggested that the retinal nerve fiber layer (RNFL) changes are associated with the presence of DPN (Shahidi et al., 2012; Salvi et al., 2016; Dehghani et al., 2017). A recent report suggested a close link between microcirculation dysfunction and DPN (Hu et al., 2021). In addition, several studies using fundus photography have shown significant retinal vascular changes in DPN patients compared with healthy controls (Ding et al., 2012; Hu et al., 2021). However, retinal photography imaging rarely gives information about deeper retinal microstructure, so subtle microvascular changes that occur at the capillary level may be missed.

OCTA is a non-invasive, non-contact imaging technology that enables the high-resolution visualization of the retinal microvasculature network in different retina layers. Cumulative reports using OCTA (Kim A et al., 2016; Chen et al., 2017) show that the retinal microvascular density of diabetic patients is lower than that of healthy controls. However, little is known about the retinal microvascular changes in DPN patients. In this study, we used an automated framework based on the state-of-the-art deep learning approach to extract retinal microvasculature to characterize the macula microvascular alterations that occur in type 2 diabetic patients with DPN and those without DPN. We also determined the ability of the deep learning approach to find the changes in the retinal microvascular network at different levels in different subareas and to look for indicators that can prompt the diagnosis of DPN patients so as to improve the diagnostic rate of DPN patients.

## Materials and methods

### Participants

This was an observational cross-sectional study. Subjects admitted to the Affiliated People’s Hospital of Ningbo University were enrolled between November 2019 and August 2021. The study was approved by the Ethics Committee of The Affiliated People’s Hospital of Ningbo University and followed the Declaration of Helsinki. All participants provided informed consent.

Diabetes mellitus patients with and without DPN and healthy controls were included in our study. Patients with type 2 diabetes mellitus were diagnosed according to WHO standards (Alberti and Zimmet 1998). A retinal specialist (YW) used a modified Airlie House classification (Wu et al., 2013) to select DM patients with retinopathy.

Basic information, such as medical history and symptoms, was collected from all participants. Besides, neurological examination of the nervous system and nerve conduction tests were also performed. Neurological examinations included pain sensation, temperature sensation, tactile sensation, vibration sensation, and ankle reflex. According to the comprehensive analysis of peripheral symptoms, signs, and nerve conduction test results (Tesfaye et al., 2010; Lipsky et al., 2012), the DPN diagnosis was confirmed by a neurologist (AC). Therefore, we classified possible and probable DPN into the NDPN group, and subclinical and confirmed DPN into the DPN group (Sloan et al., 2021).

The healthy controls were in good health in the past, with no history of diabetes and no history of eye diseases or surgery. Clinical information such as diabetes and hypertension were recorded for all participants. Controls were excluded if they had the following: trauma or toxic disorder affecting the brain, optic nerve, or retina, current or previous drug abuse, uncontrolled hypertension, hypotension, and any neuro-ophthalmic disease which could affect the retina, patients with diabetic retinopathy in the proliferative phase and non-proliferative phase in which the retinal structures were affected by bleeding, edema, and other reasons.

### Ophthalmic examinations

All the enrolled subjects underwent complete binocular examination, including best corrected visual acuity, intraocular pressure, anterior segment slit lamp examination, ultra-wide angle fundus photography, and OCTA examination. Type 2 diabetes mellitus (primary and long-term) patients were 30–80 years old, with ametropia between +3.00 D and −3.00 D, intraocular pressure between 10 and 21 mmHg, no obvious turbid refractive media, no history of glaucoma, no diffuse or focal sheath thinning, no retinal hemorrhage or macular defect, and no treatment history of active eye disease. Participants with the following conditions were excluded: 1) serious cardiovascular and cerebrovascular diseases, malignant tumors, immunological diseases, etc.; 2) history of intraocular surgery (exception: cataract extraction within 12 months); 3) ocular disease affecting the retina that has been diagnosed in the past, including macular edema, the onset of glaucoma, optic nerve disease, and choroidal neovascularization; 4) image quality affected by abnormal refractive medium or poor fixation (<8); 5) a patient with diabetic retinopathy greater than NPDR2 grade 2 or diabetic retinopathy affecting the fovea; 6) poor control of hypertension causes hypertensive retinopathy.

### OCTA imaging

Zeiss Cirrus 5000-HD-OCT Angioplex (Carl Zeiss Meditec, Dublin, CA, United States) with a scanning rate of 68,000 A-scans/s was used for retinal imaging. Each B-scan consisted of 350 A-scans in the horizontal and vertical directions. OCTA equipment includes three-dimensional projection artifact elimination (3D PAR) technology, which can minimize the artifacts in images while keeping the authenticity of the images.

Both eyes of all participants were examined and imaged. The macula was analyzed using B-scans covering an area of 3 × 3 mm^2^ repeated horizontally and vertically. Images of good quality (signal quality≥8) were selected for further analysis. Angiograms with irregular patterns of vessels or irregular vascular segmentation were excluded. Retinal layer segmentation of the macula was done commercially by an inbuilt algorithm in the OCTA tool (Spaide et al., 2014). The retina in the macular region was divided into the superficial vascular complex (SVC, from the internal limiting membrane to 10 μm above the inner plexiform layer) and deep vascular complex (DVC, from 10 μm above the inner plexiform layer to the 10 μm below the outer plexiform layer) for microstructure analysis.

### Deep learning algorithm on macular microvasculature

A state-of-the-art OCTA-Net algorithm was used for the microvasculature segmentation (Ma et al., 2020). To construct this model, we used a coarse-to-fine segmentation method, in which the initial confidence map for the retinal microvascular network was generated first, followed by the outline of the macular microvasculature. The OCTA-Net was trained on the public OCTA Segmentation Dataset (ROSE); its effectiveness has been well documented in a previous report (Ma et al., 2020). Vascular length density (VLD) was used to assess the macular microvasculature; VLD is defined as the ratio of the total number of pixels on microvascular centerlines to the measurement area. VLD in the SVC and the DVC was calculated using MATLAB software.

**FIGURE 1:**
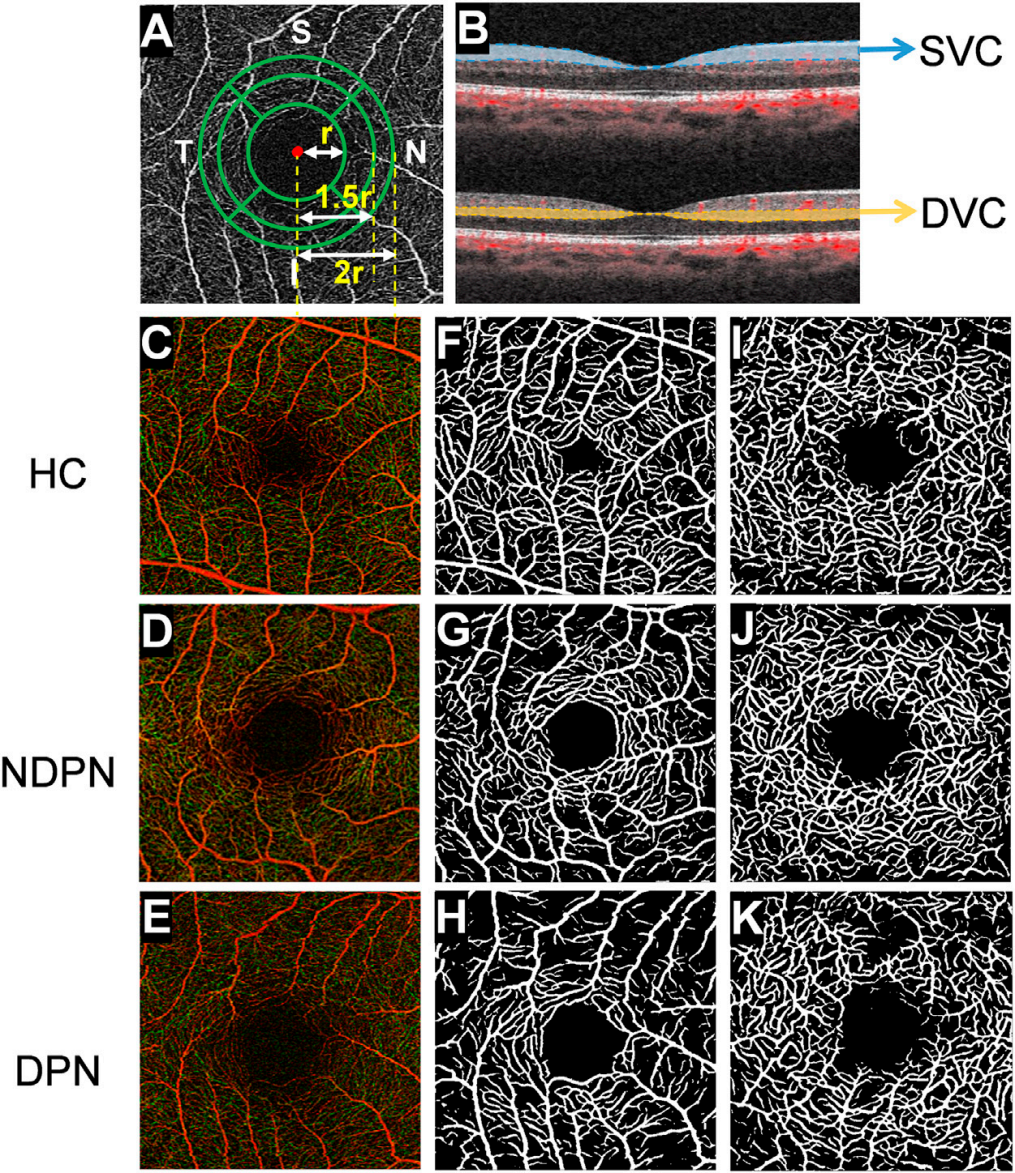
OCTA image analysis of macular fovea and artificial intelligence algorithm for layered analysis of images. **(A)** Using OCTA to scan the macula area of the subject within the range of 3 × 3 mm^2^, and the images of capillaries around the fovea are obtained. Then, using deep learning software, FAZ was used to fit a circle with radius r, after which three concentric circles with radius r, 1.5 r, and 2 r were drawn. With further partitioning, the retina was divided into inner and outer rings: nasal area, superior area, temporal area, and inferior area, and there were 8 areas in total. **(B)** The retina was segmented into SVC (from the internal limiting membrane to 10 μm above the inner plexiform layer) and DVC (from 10 μm above the inner plexiform layer to the 10 μm below the outer plexiform layer) images with different depths. The images **(C–K)** show retinal microvascular in a different layer of HC, NDPN, and DPN groups. **(C–E)** The images show the full-thickness retinal microvasculature produced by OCTA in HC, NDPN, and DPN groups. **(F–H)** Capillaries in the SVC by deep learning software. **(I–K)** Capillaries in the DVC by deep learning software.

The VLD value of the nine quadrant sectors (center, superior inner, temporal inner, inferior inner, nasal inner, superior outer, temporal outer, inferior outer, and nasal outer) was analyzed to give the mean value. To generate the sub-sectors, we first fit a circle with radius r using the foveal avascular zone (FAZ) and then drew three concentric circles with radius r, 1.5 r, and 2r, respectively, as shown in Figure 1 to exclude the potential influence of the FAZ (Figure 1).

### Statistical analysis

IBM SPSS Statistics 23 software (version 23; SPSS, Inc. Chicago, IL, United States) was used for statistical analysis. The Kolmogorov-Smirnov test was applied to assess the normality of the data. Quantitative variables were expressed as mean ± standard deviation (SD). Refraction data were converted to spherical equivalent (SE). The best corrected visual acuity was expressed as the logarithm of the minimum resolution angle (LogMAR). Generalized estimating equations (GEE) were used to assess the differences among the groups while adjusting for age, gender, mean arterial pressure, and signal quality of angiograms. The subjects’ receiver operating characteristic (ROC) curve was used to assess the diagnostic capability of the VLD calculated by the artificial intelligence recognition system for diabetes with DPN. Larger areas under the ROC curve (AUROC) indicated higher diagnostic values. A *p*-value <0.05 was considered statistically significant.

## Results

### Demographic characteristics among three groups

We initially enrolled 30 healthy controls and 66 DM patients; three healthy controls were excluded due to age macular degeneration (AMD) and three DM patients because of uncooperativeness during retinal imaging using the OCTA. Finally, 27 healthy controls (42 eyes), 36 DM patients without DPN (62 eyes), and 27 DM patients with DPN (40 eyes) were included in data analysis. There was no significant difference among the three groups in age, sex, and MAP (all *p* > 0.05, Table 1). Yet, there was a significant difference in visual acuity between the three groups (*p* = 0.003, Table 1) and the visual acuity was significantly lower in the DPN group vs the other two groups. As far as the duration of diabetes is concerned, the disease duration in the DPN group was obviously longer than that of the NDPN group (*p* = 0.043, Table 1).

**TABLE 1:** Demographic characteristics of all subjects.

Parameters	Control, *n* = 27	NDPN, *n* = 36	DPN, *n* = 27	p
Eyes	42	62	40	
Age, y	57.12 ± 13.36	55.50 ± 10.11	59.63 ± 8.53	0.324
Sex, M/F	10:17	22:14	16:11	0.129
BMI	21.97 ± 1.68	24.50 ± 2.92	22.84 ± 3.61	0.003
MAP, mmHg	91.69 ± 7.12	92.44 ± 8.97	91.99 ± 8.98	0.944
SE, diopter	−0.41 ± 1.10	−0.29 ± 1.72	−0.12 ± 0.93	0.628
BCVA, logMAR	0.01 ± 0.03	0.01 ± 0.04	0.06 ± 0.13	0.003
IOP, mmHg	15.40 ± 2.89	15.83 ± 2.90	16.74 ± 3.70	0.148
Duration, y	NA	6.40 ± 6.81	9.93 ± 6.47	0.043
HbA1c	—	9.75 ± 2.19	9.36 ± 2.14	0.499
BG	—	9.36 ± 3.55	9.55 ± 4.19	0.843
TG	—	2.18 ± 2.90	2.12 ± 1.54	0.917
T-CHOL	—	4.83 ± 1.27	4.53 ± 1.48	0.388
HDL-C	—	1.15 ± 0.32	1.07 ± 0.30	0.272
LDL	—	3.01 ± 0.75	2.77 ± 1.04	0.314

Values for continuous variables are means ± standard deviations for all subjects in each group. HC, healthy control; NDPN, Diabetic patients without DPN; DPN, diabetic patients with peripheral neuropathy; –, not performed; NA, not applicable; BCVA, best-corrected visual acuity. One-way ANOVA, for numerical data.

### Changes in VLD among the three groups

To further explore the correlation between DPN and microvascular changes in the retina, we used OCTA to collect fundus blood vessels from three groups of subjects and used the intelligent recognition system trained on the public ROSE dataset to segment the vascular. There was no significant difference in the average VLD values of the SVC (*p* = 0.868, Table 2) and DVC (*p* = 0.286, Table 2) in NDPN when compared with healthy controls; yet, DPN patients showed significantly lower SVC (*p* = 0.010, Table 2) and DVC (*p* = 0.011, Table 2) average VLD values when compared with healthy controls. Also, compared with NDPN, DPN showed significantly lower SVC (*p* = 0.006, Table 2) and DVC (*p* = 0.001, Table 2) average VLD values.

**TABLE 2:** Comparison of VLD value among the three groups.

		HC	NDPN	DPN	P1	P2	P3
SVC							
Average		7.61 ± 0.73	7.50 ± 0.84	6.89 ± 0.98	0.868	0.010	0.006
Inner	Nasal	11.38 ± 0.83	10.90 ± 1.19	10.35 ± 1.04	0.04	<0.001	0.023
	Superior	11.47 ± 1.03	11.16 ± 0.98	10.30 ± 1.17	0.311	<0.001	<0.001
	Temporal	11.10 ± 1.02	10.79 ± 1.06	10.22 ± 1.21	0.203	0.002	0.033
	Inferior	11.63 ± 1.07	11.32 ± 1.17	10.70 ± 1.22	0.189	0.001	0.019
Outer	Nasal	25.01 ± 2.59	24.76 ± 3.03	23.12 ± 2.78	0.499	0.003	0.007
	Superior	24.93 ± 3.15	24.61 ± 3.51	22.70 ± 3.16	0.918	0.009	0.005
	Temple	24.33 ± 3.08	24.16 ± 2.70	23.23 ± 3.09	0.805	0.169	0.198
	Inferior	24.29 ± 3.16	24.35 ± 3.15	23.33 ± 3.56	0.97	0.219	0.181
DVC							
Average		8.07 ± 0.41	8.07 ± 0.63	7.60 ± 0.57	0.286	0.011	0.001
Inner	Nasal	9.02 ± 1.74	9.07 ± 1.60	8.84 ± 1.67	0.985	0.506	0.448
	Superior	11.02 ± 0.97	10.87 ± 1.26	10.19 ± 1.24	0.554	0.003	0.006
	Temporal	9.38 ± 1.47	9.12 ± 1.65	9.11 ± 1.63	0.426	0.452	0.962
	Inferior	10.64 ± 1.40	10.48 ± 1.70	10.32 ± 1.54	0.6	0.354	0.607
Outer	Nasal	28.92 ± 2.42	28.37 ± 3.11	27.43 ± 3.00	0.336	0.056	0.237
	Superior	28.45 ± 2.22	28.55 ± 2.80	26.48 ± 3.01	0.796	0.002	<0.001
	Temple	28.89 ± 2.29	28.71 ± 3.31	27.09 ± 4.09	0.738	0.037	0.046
	Inferior	28.47 ± 2.47	28.06 ± 3.10	26.84 ± 3.24	0.69	0.065	0.096

P1: comparison between HC, and NDPN; P2: comparison between HC, and DPN; P3: comparison between NDPN, and DPN., data were adjusted for age, gender; MAP, and signal quality of angiograms; SVC, superficial vascular complex; DVC, deep vascular complex.

**FIGURE 2:**
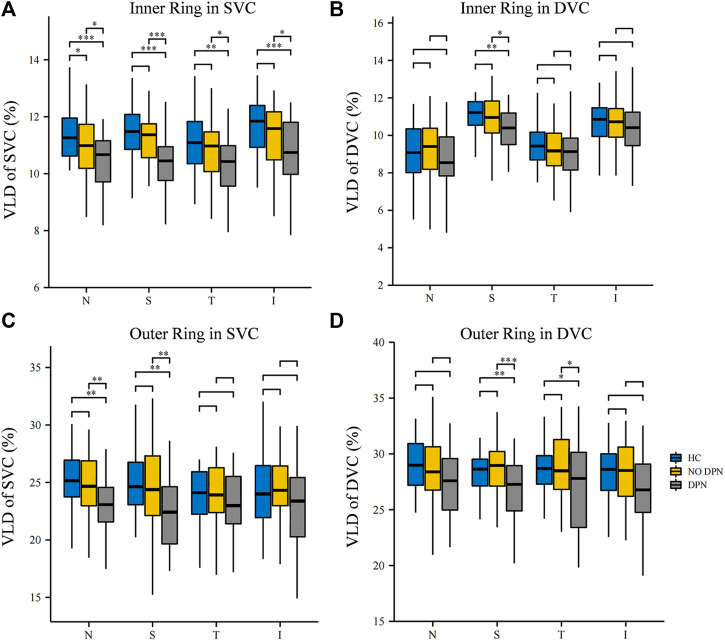
Comparison of VLD in different sectors between HC, NDPN, and DPN groups. **(A)** Describe the VLD of the SVC in four inner quadrant sectors (nasal inner, superior inner, temporal inner, and inferior inner). **(B)** VLD of DVC in four inner sectors. **(C)** VLD of SVC in four outer quadrant sectors (nasal outer, superior outer, temporal outer, and inferior outer). **(D)** VLD of DVC in four outer quadrant sectors. VLD is defined as the ratio of the total number of pixels on microvascular centerlines to the area of measurement.

Next, the VLD values of the SVC and DVC in the eight sections around the fovea were compared among the three groups. Figure 2 shows the comparison of VLD values in the eight sections of the SVC and DVC among the three groups. DPN patients showed significantly lower VLD values (*p* < 0.05, Figure 2) in the nasal, superior, temporal and inferior sectors of the inner ring of the SVC when compared with controls; likewise, VLD values in NDPN patients were significantly lower in the nasal section of the inner ring of the SVC (*p* < 0.05, Figure 2) compared with controls. Importantly, DPN patients showed significantly lower VLD values (*p* < 0.05, Figure 2) in the four inner ring sections of the SVC compared with NDPN. As for DVC, we found that the VLD values coming from the DPN group were significantly decreased in superior sectors of the inner ring, as well as superior and temporal sectors of the outer ring (*p* < 0.05, Figure 2) when compared with healthy controls and NDPN groups.

**FIGURE 3:**
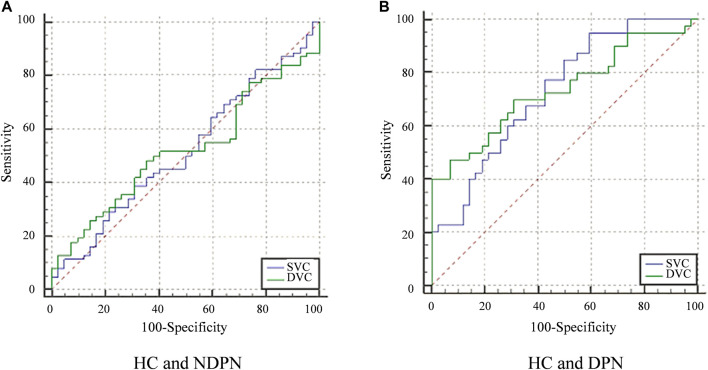
ROC curve analysis of VLD in the SVC and DVC. **(A)** The diagnostic efficiency of VLD value of NDPN group in different levels. **(B)** The diagnostic efficiency VLD value of the DPN group. Greenline: The ROC of DVC. Blueline: The ROC of SVC.

ROC curve analysis was carried out to determine the ability of VLD to detect alterations in microvascular of DPN and NDPN (Figure 3). The VLD values in the DVC of the DPN group showed a higher ability to discriminate microvascular damage when compared with the NDPN group (Figure 3). The results showed that the area under the curve (AUC) of SVC in the diagnosis of DPN was 0.729 (*p* < 0.001), while the cutoff point of 8.01 showed a sensitivity of 95%, a specificity of 40.48% (Table 3). On the other hand, the AUC of DVC for DPN development was 0.736 (*p* < 0.001), with a cutoff point of 7.47, showing a sensitivity of 47.50% and a specificity of 92.86%. SVC (AUC = 0.519, *p* > 0.05) and DVC (AUC = 0.520, *p* > 0.05) had poor diagnostic values for NDPN (Table 3).

**TABLE 3:** ROC curve analysis of VLD in different layers of DM with or without DPN.

	AUC (95%CI)	Cutoff	Sen, %	Spe, %	*p*
NDPN					
SVC	0.519	7.41	38.71	69.05	0.740
DVC	0.520	8.18	48.39	64.29	0.721
DPN					
SVC	0.729	8.01	95.00	40.48	<0.001
DVC	0.736	7.47	47.50	92.86	<0.001

AUC, area under the curve; CI, confidence interval; Cut Off, the magnitude of the analyte to be detected; Sen, sensitivity; Spe: specificity.

## Discussion

It would be beneficial to gain a better understanding of the neurodegeneration and vascular complications in both normal and DPN patients in order to determine the characteristics and progression of this disease. Traditional neurodegenerative indicators are not sensitive enough to detect early DPN and identify disease progression (Iqbal et al., 2018). As a result, half of the diabetic patients have DPN in the course of their disease development, but only about 20% of them have typical clinical manifestations of neuropathy at the time of diagnosis (Deli et al., 2013; Hosseini and Abdollahi 2013; Verrotti et al., 2014). Our research provides a new method for diagnosing DPN, which is expected to become an objective biomarker for predicting which patients may develop from NDPN to DPN in the future.

Previous cross-sectional studies (Ding et al., 2012; Hu et al., 2021) showed that DPN patients have a retinal microvascular abnormality, which is not seen in healthy controls. However, these reports used fundus photography, which limits the resolution of the retinal microvasculature to the superficial vessels. In this study, we used OCTA to image the macular microvasculature and utilized a deep learning approach to assess the VLD in DPN and NDPN when compared with healthy controls and determined the diagnostic ability of the deep learning approach to identify the macular capillary changes. DPN was significantly altered in SVC and DVC when compared with healthy controls and NDPN. The SVC layer is responsible for the metabolic supply of the ganglion cell. A previous study observed degeneration of retinal ganglion cells and axons in SVC in diabetic patients (Kim K et al., 2016; Kim et al., 2018). Changes in the SVC seen in our report complement the already reported OCT structural markers (Shahidi et al., 2012; Sangeetha et al., 2016). On the other hand, DVC lies beneath the SVC and is important for the nutrition of the inner nuclear layer. This microvascular plexus consists of bipolar cells, horizontal cells, and amacrine cells found in the deeper portion of the inner retina. The DVC is supplied by vertical anastomoses of the SVC (Campbell et al., 2017), indicating that changes in the SVC may affect the DVC. The microvascular changes seen in our current study are in line with previous OCTA studies (Nesper et al., 2017; Conti et al., 2019; Dai et al., 2020), which used different quantitative programs to characterize the microvascular changes suggesting that peripheral neuropathy may lead to microvascular impairment.

Patients with DM can exhibit microvascular damage (Thrainsdottir et al., 2003), endothelial cell proliferation, and intimal thickening, which results in blockage of the vessel lumen, ultimately leading to hypoperfusion and dysfunction of nerve cells (Malik et al., 1993). Microvascular impairment progresses into neuropathy and is one of the hallmarks of DPN (Gibbons and Shaw 2012). In the early phase, microvascular impairment and peripheral neuropathy are principally asymptomatic, and few tests are accessible for diagnosis. Interestingly, we found that the VLD in DPN patients’ nasal and superior areas was more susceptible and pronounced than in other areas. Similarly, Radi *et al.* found that the retina vessel density in the superficial of the macular region was significantly reduced, and the decrease in the superior and temporal sectors was the most obvious in the early stage of DR (Radi et al., 2019). In addition, Li et al. (2020) performed fundus angiography and fundus photography on patients with diabetic retinopathy at different stages and found that the exudation and microvascular lesions on the nasal side of the posterior pole increased significantly in the early phase of diabetic retinopathy and the microvessels on the nasal side were significantly damaged. However, some scholars found that diabetic microvascular abnormalities were more common on the temporal side than on the nasal side in the early stage of retinopathy in diabetic patients (Silva et al., 2013; Matsunaga et al., 2015). This phenomenon of uneven distribution of lesions may be related to the uneven physiological or metabolic processes in different regions, such as abnormal expression and distribution of biochemical substances such as caspase-1 (Glut1), and inducible nitric oxide synthase (iNOS) and PKCE (Tang et al., 2003). Other scholars believe that the differences in microvascular changes in different sections may be related to the different anatomical structures of retinal microcirculation (Chaher et al., 2022). However, the specific mechanism is still unclear.

Importantly, we analyzed the ability of the macular microvasculature density to detect the early changes in DPN and NDPN groups. Our ROC curve analysis showed the ability of VLD in SVC and DVC to discriminate between HC and DPN, HC, and NDPN; however, the DVC showed a higher discriminating power than the SVC. Noteworthy, the DVC has a thinner and smaller microvascular structure making it more sensitive to the progression of the disease than the SVC (Wang et al., 2018); thus, we suggest that this plexus may be more sensitive to the microvascular damage associated with the disease cascade. This implies a close connection between neurodegeneration and microvasculature in these patients. Since retinal microvascular has been suggested to reflect microvascular diseases in other parts of the body (Cankurtaran et al., 2020; Zhang et al., 2021), we suggest that assessment of retinal microvasculature could be a route for the early detection of microvascular degenerates as indirect pointers of DPN. Such *in vivo* quantitative means allow monitoring of DPN and may enable the assessment of treatments. Therefore, endocrinologists should comprehensively consider the retinal structure and microvasculature in estimating and treating the early DPN of DM patients.

There are several limitations in the present study. First, six participants were excluded due to movement during OCTA imaging. Moreover, healthy control subjects were not examined for HbA1c. Although the history of past medical conditions was obtained from each patient, we cannot rule out the possibility of underlying diseases. Second, this cross-sectional study did not comprehensively analyze retinal microvascular parameters over time and disease progression in patients with DPN. Finally, the sample size is relatively small. The relationship between the development of retinal vascular changes over time and DPN should be further explored through a multi-center longitudinal study with larger samples.

## Conclusion

Our results showed that DM patients with DPN had significantly lower SVC and DVC VLD, and the VLD in the nasal and superior sectors of DPN patients was more susceptible and more pronounced. We also found that the AUCs for VLD of the SVC and DVC could discriminate between DPN patients and controls to a certain extent and may serve as an early pointer of microangiopathy. OCTA based on deep learning could be potentially used in clinical practice as a new indicator in the early diagnosis of DM with and without DPN.

## Data Availability

The raw data supporting the conclusion of this article will be made available by the authors, without undue reservation.
